# Comparison of Depth Camera and Terrestrial Laser Scanner in Monitoring Structural Deflections

**DOI:** 10.3390/s21010201

**Published:** 2020-12-30

**Authors:** Michael Bekele Maru, Donghwan Lee, Kassahun Demissie Tola, Seunghee Park

**Affiliations:** 1 Department of the Civil, Architectural and Environmental System Engineering, Sungkyunkwan University, Suwon 16419, Korea; mikemaru@skku.edu (M.B.M.); kastolla@skku.edu (K.D.T.); 2 Department of Convergence Engineering for Future City, Sungkyunkwan University, Suwon 16419, Korea; ycleedh@gmail.com; 3 School of Civil, Architectural Engineering and Landscape Architecture, Sungkyunkwan University, Suwon 16419, Korea

**Keywords:** terrestrial laser scanning (TLS), depth camera (DC), hausdorff distance, bilateral filtering, point cloud, deflection

## Abstract

Modeling a structure in the virtual world using three-dimensional (3D) information enhances our understanding, while also aiding in the visualization, of how a structure reacts to any disturbance. Generally, 3D point clouds are used for determining structural behavioral changes. Light detection and ranging (LiDAR) is one of the crucial ways by which a 3D point cloud dataset can be generated. Additionally, 3D cameras are commonly used to develop a point cloud containing many points on the external surface of an object around it. The main objective of this study was to compare the performance of optical sensors, namely a depth camera (DC) and terrestrial laser scanner (TLS) in estimating structural deflection. We also utilized bilateral filtering techniques, which are commonly used in image processing, on the point cloud data for enhancing their accuracy and increasing the application prospects of these sensors in structure health monitoring. The results from these sensors were validated by comparing them with the outputs from a linear variable differential transformer sensor, which was mounted on the beam during an indoor experiment. The results showed that the datasets obtained from both the sensors were acceptable for nominal deflections of 3 mm and above because the error range was less than ±10%. However, the result obtained from the TLS were better than those obtained from the DC.

## 1. Introduction

The demand for structural steel has been increasing over time owing to its numerous benefits, such as high ductility and tensile strength, lighter weight relative to concrete, and ease of use in construction. During its service life, its performance may be affected by unintended excessive impact loads and environmental disasters. These and several other defects lead to unforeseen structural damages, resulting in major catastrophes. To solve this problem, a structural health monitoring technique is required from the early construction period to the end of its service life, depending on the service levels. Essentially, structural health monitoring should be conducted in two phases. The first phase monitors the structural response for any load during the service time using permanently mounted sensors [1]. The sensor that is affixed to the structure should be inexpensive because economic considerations are an important aspect in the construction industry and should be optimized. Optical sensors, such as depth cameras (DCs), kinetic cameras, fiber optics sensors, and range cameras, are the most favorable inexpensive vision-based sensors and are effective in acquiring structural responses via images, videos, and spatial coordinates [2,3,4]. The second phase should be carried out before a certain period of time to account for any changes in the physical shape of the members that may occur [5]. Accordingly, depending on the technique, an adequate budget must be allocated before utilizing this phased approach. Terrestrial laser scanners (TLSs), airborne laser scanners (ALSs), and unmanned aerial vehicles (UAVs) are the most popular and valuable means of capturing structural real-time 3D shapes that are vital input data for structural health assessment [6,7,8]. Vision-based sensors are used for quality and process control in various commercial and research applications, as well as medical technologies, automotive industries, metrology, and remote sensing [9,10,11]. Meanwhile, different types of optical sensors have been used to monitor structural health.

Several methods have been proposed for using vision-based sensors. Specifically, a component of the serviceability limit state of a structure should be given attention and monitored periodically using different types of noncontact optical sensors. Among these components, some researchers have investigated the dynamic displacement of a structure using a range camera [12], TLS [13], and Microsoft Kinect [14]. Crack detection and measurement, structural vibration, and real-time structural 3D modeling have also been accomplished using TLS and a camera [4,15]. Structural deflection due to creep, unexpected loading, and different environmental impacts is a crucial serviceability limit state behavior of a structure. Gordon and Lichti estimated beam deflection using point cloud data derived from TLS acquired at different epochs and compared the results by extracting a line directly from the point cloud data as a reference [16]. Park et al. estimated structural element (beam) deflection and its corresponding maximum stress using the same approach [7]. However, the line is derived from the representative fitted plane of the point cloud for setting a new coordinate system, which helps evaluate the real magnitude of structural shape changes. To improve the deflection estimation results obtained Park et al., a group of researchers obtained the curve of a beam deflected shape using a heuristic process called a genetic algorithm to obtain a better deflection value [17]. Although the RealSense DC is not yet optimized for structural health monitoring, Sayyar-Roudsari proposed an inspection method to detect threats to a defective beam produced by honeycomb structural components [18]. Some researchers have also attempted to fuse image depth data with RGB data to increase the performance of vision-based depth sensors using data fusion techniques [19]. Other researchers also used two different types of vision-based sensors involving data fusion techniques to generate a robust approach and produce satisfactory results [20].

The main goal of this research was to develop a technique that can measure the deflection of a beam structure from light detection and ranging(LiDAR) and DC data individually. In addition, the results were compared to determine the impact of these methods on structural health monitoring. Through this study, we aimed to determine the performance levels of inexpensive vision-based cameras as alternatives to expensive and complicated sensor systems in structural health monitoring. Evaluating the performance of these sensors is vital for performing quality control at low cost. In addition, we examined the performance of bilateral filtering on optical and/or vision-based sensor data for better implementation of structural health monitoring techniques. Section 2 describes the data preparation process and denoising techniques used for the sensor data. Section 3 provides an overview of the two evaluated sensors and describes the developed methodology for each TLS and DC followed by the design of the experimental setup. An indoor laboratory test was performed with a series of loadings to obtain the nominal deflection. In Section 4, the data processing methods for both vision-based sensors and their outcomes are discussed. Finally, the validated results obtained from the two sensors are compared and the major findings of the study are summarized.

## 2. Data Preparation

### 2.1. Data Acquisition and Pre-Processing

The data were simultaneously acquired through optical sensors, an Intel Real sense DC, and a Leica TLS. For the TLS case, the highest resolution mode was used. This mode of resolution has a capacity of holding 2530 × 2181 points in the horizontal and vertical directions based on Leica specifications. One of the limitations of using this mode is having a large amount of data, which makes post-processing difficult. However, our specimen is small compared to the capacity of nature of TLS. Therefore, using a high-resolution mode of scanning has better output performance. Considering the geometric shape of the specimen, range, and surrounding environment, we used an effective field of view before starting the scanning process. We scanned the specimen three times during each loading case to ensure safety. The scanning process started immediately after applying the corresponding load to attain the nominal deflection. when using the DC, real-time data acquisition via a LibRealSense SDK2.0 platform was conducted. This platform provides a RealSense Viewer application for users to access most camera functionalities through a simple cross-platform user interface. Streaming from multiple RealSense devices at the same time, exploring point cloud data in real time, recording and playback of the recorded RealSense device data, and access to most camera-specific controls are some of the benefits offered by the viewer application. As with TLS, the RealSense data were recorded immediately after applying the nominal load and attaining the deflection.

Pre-processing of the point cloud data is involves preparing the data before proceeding to the data processing step [21]. LiDAR point cloud data inputs to tools found in the surrounding must be pre-processed to remove outliers and/or noise, errors, and non-target data points through manual editing. To decrease the errors resulting from manual cropping of the targeted area from the raw data, we processed the data multiple times in each case and took the average.

### 2.2. Denoising

When acquiring point cloud data, owing to the impacts of equipment accuracy, operator experience, environmental conditions, and other factors, some noise points (that is, points we cannot use) are inevitable in point cloud data. We need to remove these points before processing the data. Outlier and noise removal(denoising) in pre-processing is interpreted differently by many researchers [22]. However, to the best of our knowledge, outlier removal uses a noise removal filter that permits deletion of the lonely points outside a detected surface, whereas the noise remover filter removes points that do not match the local geometrical behavior of the point cloud. Specifically, data from both sensors were subjected to different denoising techniques for better output. The following two subsections describe the techniques we used on raw data before processing it.

#### 2.2.1. Interquartile Range

Outliers are part of the data that are distant from other observations. Although outliers are unknown in the acquisition phase, they may result from result from errors during data collection and indicate variance in our data. In a vast database, differentiating the outliers from the actual data is challenging. Therefore, researchers have been trying to manipulate outliers in a systematic manner in combination with the statistical behavior of the data. Among the numerous methods of treating outliers, the interquartile range (IQR) score is a crucial and robust method. The IQR plays an important role in data science, for removing outliers from a set of data [23]. The IQR is similar to the Z-score in terms of finding the distribution of data and maintains a threshold to identify the outlier. In statistics, a simple way of knowing the distribution of data is by calculating the range, which can be obtained by deducting the minimum value from the maximum value. The IQR is calculated in the same way as the range. However, the maximum and minimum data are replaced by the third quartile and first quartile, respectively, as shown in Equation (1).

(1) $$IQR=Q_{3}-Q_{1}.$$

**Figure 1:**
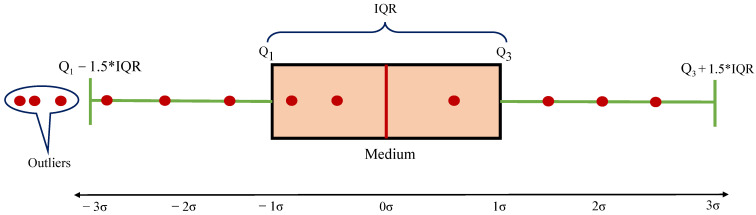
Interquartile range (IQR) configuration.

After arranging the data in an ascending order, $Q_{1}$ represents a quarter of the way through the data list, whereas $Q_{3}$ represents three-quarters of the way through the data list. The interquartile range shows how the data are spread about the median. It is less susceptible than the range to outliers. Figure 1 shows an overview of the IQR process.

The following are the steps involved in the detection and removal of outliers using IQR:

- Maintaining the data in ascending order.
- Obtaining the median values, $Q_{1}$, and $Q_{3}$, used in determining the interquartile range.
- Scaling the interquartile range by 1.5.
- Adding and deducting the achieved value onto $Q_{3}$ and $Q_{1}$, respectively.
- Removing the set of data beyond these two ranges.

#### 2.2.2. Bilateral Filter

Bilateral filtering techniques are notable image denoising methods, first conducted by Tomasi and Manduchi [24]. Technically, bilateral filtering is a modified version of Gaussian smoothing. However, unlike Gaussian smoothing and average filtering, it preserves the sharp edges of an image during noise removal. The bilateral filter varies with each pixel of an image by a weighted average of its neighbor, based on the spatial distance within the pixels and the similarity between color samples. This filter is formulated as follows:

(2) $$p^{{}^{\prime }}=p+\gamma \times n_{i},$$

where $\gamma =\frac{\underset{q_{ij}\in Q_{r}}{\Sigma }w_{c}(∥p_{i}-q_{ij}∥)w_{s}\left(<n_{i},p_{i}-q_{ij}>\right)<n_{i},p_{i}-q_{ij}>}{\underset{q_{ij}\in Q_{r}}{\Sigma }w_{c}(∥p_{i}-q_{ij}∥)w_{s}\left(<n_{i},p_{i}-q_{ij}>)\right)}$ is a bilateral filtering weight.

*p* is the noisy point from TLS or DC.
$p^{{}^{\prime }}$ is the denoised point of *p*.
$n_{i}$ is the normal vector of point *p*.
$w_{c}$ is the 2D Gaussian filter for smoothing.
$w_{s}$ is the 1D Gaussian weight function for preserving edge features.
$q_{ij}$ is a neighborhood point within the distance range, r, from *p*.
$g=∥p_{i}-q_{ij}∥$ is the geometric distance between *p* and $q_{ij}$.
$h=<n_{i},p_{i}-q_{ij}>$ is an inner product between the normal of a point, *p*, and the geometric distance, *g*.
$\sigma_{c}$ & $\sigma_{s}$ are parameters defined as the standard deviation of the neighborhood distance of point *p* and a factor of the projection of the distance vector from point *p* to its neighborhood point on the normal vector, $n_{i}$, of point *p*, respectively.

This technique has been applied for image smoothing involving meshes and point clouds by different researchers at different times [25,26]. To the best of the our knowledge, Lihui Wang and his research team are the first to conduct bilateral filtering for denoising of point clouds [27]. They applied fuzzy c-means (FCM) clustering before bilateral filtering to diminish the large-scale noise in the data. However, bilateral filtering is incapable of denoising large-scale noises. Consequently, our DC dataset was overdosed for this specific denoising technique. Therefore, we applied IQR techniques to diminish the large-scale noise. After determining the principle of bilateral techniques, the widely accepted form of pseudocode representation is given as follows: Algorithm 1.
**Algorithm 1.** Bilateral denoising $\left(p,r,\sigma_{c},\sigma_{s}\right)$**Input**: points from TLS and/or DC obtained from deflected and/or undeflected beam, p$Q_{r}\left(p\right)$ ← neighborhood of a selected point, p, within radius r of surroundings.Evaluate the unit normal vector, $n_{i}$, to the regression plane, $n_{p}$, from $Q_{r}\left(p\right)$**Output**: denoised point p’**        For**$q_{ij}\;\epsilon \;Q_{r}$**do**,$\;\;\;\;\;\;\;\;\;g=∥p_{i}-q_{ij}∥$$\;\;\;\;\;\;\;\;\;h=<n_{i},p_{i}-q_{ij}>$$\;\;\;\;\;\;\;\;\;w_{c}=e^{-g^{2}⁄2{\sigma_{c}}^{2}}$$\;\;\;\;\;\;\;\;\;w_{s}=e^{-h^{2}⁄2{\sigma_{s}}^{2}}$$\;\;\;\;\;\;\;\;\;\gamma =\frac{\underset{q_{ij}\in Q_{r}}{\Sigma }w_{c}(∥p_{i}-q_{ij}∥)w_{s}\left(<n_{i},p_{i}-q_{ij}>\right)<n_{i},p_{i}-q_{ij}>}{\underset{q_{ij}\in Q_{r}}{\Sigma }w_{c}(∥p_{i}-q_{ij}∥)w_{s}\left(<n_{i},p_{i}-q_{ij}>)\right)}$$\;\;\;\;\;\;\;\;\;p^{{}^{\prime }}=p+\gamma *n_{i}$**End**

## 3. Experimental Study

### 3.1. Instrumentation

#### 3.1.1. Depth Camera

**Figure 2:**
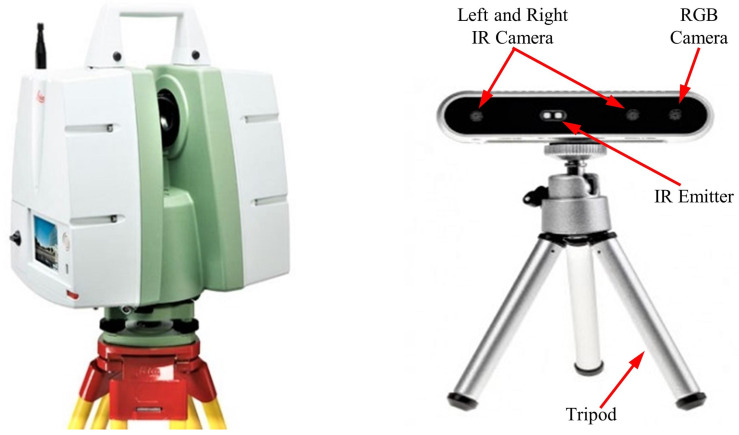
Pictorial representation of the terrestrial laser scanner (TLS) and depth camera (DC) used in this study.

A DC uses infrared light to detect the depth difference of an object relative to its coordinate system. Till date, the depth of the scene can be perceived throughout the camera either using structured light, time of flight (ToF), or stereo vision principles [28]. Principally, a structured light method projects a sequence of coded patterns on an object to determine its depth. Treating these pattern as temporal textures rather than as known codes allows multiple structured light systems to be used together. In the ToF principle, the system measures the distance based on the known speed of light, measuring the ToF of a light signal between the camera and the object for each point on the image. Because each pixel encodes the distance to the corresponding point in the scene, the depth map for the entire field of view can be produced. The stereo vision principle mimics the synchronizations between the human eyes and brain to detect the depth of an object. Projecting infrared light onto a scene improves the accuracy of the collected data. Having a constant gap between the two sensors enabled us to visualize and quantify the depth information of the scene. We used an Intel^®^ RealSense™ D415 DC based on infrared stereo vision technology in this study. Table 1 and the right side of Figure 2 provide the specifications of the DC and its pictorial representation, respectively; the camera was utilized per Intel RealSense instructions [29].

#### 3.1.2. Terrestrial Laser Scanning

TLS is a ground-based LiDAR method that scans an object by emitting a laser pulse and records the subsequent intensity of the signal returning from the object. Green (most of the time) and red visible light components from the electromagnetic spectrum are utilized in this method. Point clouds can be produced directly using a 3D scanner that records several points returning from the external surfaces of objects on which the laser light is incident. These sets of points have multidimensional information about the scanned object [30,31]. Point clouds are used for many purposes, including creating 3D models for manufactured or hand-crafted parts and objects, for quality inspection in geomatic applications, and for a multitude of visualization, animation, rendering, and mass customization applications. Lidar technology involves either phase-based, i.e., ToF, or triangulation (rare case) methods. Researchers have tried to compare the principles of available laser scanning technologies [32,33]. A ToF scanner measures the time it takes for the emitted pulse to reflect back to the scanner. The proportion of the pulse returning to the scanner depends on the object roughness. Because the emitted pulse travels at the speed of light, the distance can be obtained using the speed versus distance formula. Recent developments in this technology allow us to acquire information about the spatial position (x,y,z) of a target structure, and the intensity provides information regarding the object roughness using the energy of the returning pulse and color (RGB). RGB data are captured either through an internal or external camera and is depend on the type of camera used. Most ToF scanners are capable of measuring tens of thousands of points per second at distances of approximately 1 km. These scanners are typically used in surveying and environment scanning with a range of 5–300 m [34,35]. The left side of Figure 2 shows the Leica C5 scanner employed in this study; this scanner acquires point cloud data based on the ToF principle. Table 1 provides the specification for the Leica terrestrial laser scanner and Intel RealSense depth camera per the Leica geosystem specification manual [36].

### 3.2. Proposed Approach for Estimating Structural Deflection Using via TLS and DC

**Figure 3:**
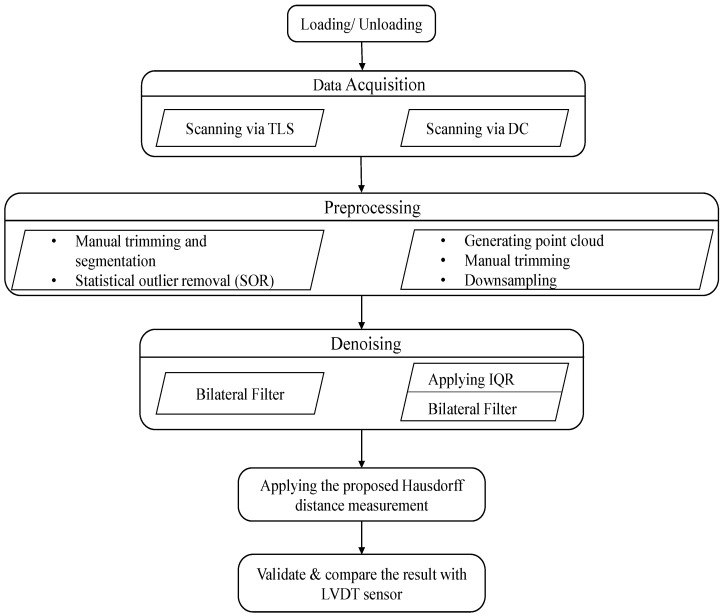
Flowchart depicting the process of comparing the results obtained using DC and TLS.

The flowchart in Figure 3 shows the generalized process of individually computing structural deflection using vision-based sensors. Once the artificial deflection is induced in the prepared specimen, both the scanning processes were conducted separately. The results were then compared based on their outputs relative to a contact sensor and a linear variable differential transformer (LVDT), mounted during the experiment.

#### Hausdorff Distance

This is the distance used to measure the difference between two subsets in a metric space. It is defined as the greatest of all the distances from a point in one set to the closest point in the other set. The concept behind it is measuring the similarity between two sets. This means that if two sets have a small Hausdorff distance, they are expected to look almost identical. It is a robust scheme to measure the distance between two points in the presence of outliers [37,38]. Given two point sets $A=a_{1},a_{2},\;...,\;a_{n}$ and $B=b_{1},b_{2},\;...,\;b_{m}$, the Hausdorff distance from A to B is a maxi-min function, which is defined in Equation (3):

(3) $$\delta_{h}\left(A,B\right)=\max_{a\epsilon A}\left\{\min_{b\epsilon B}\left\{∥a-b∥\right\}\right\},$$

where ∥a−b∥ is the underlying norm on points A and B (usually taken as the Euclidean distance). Further, a and b are points in set A and B, respectively.

**Figure 4:**
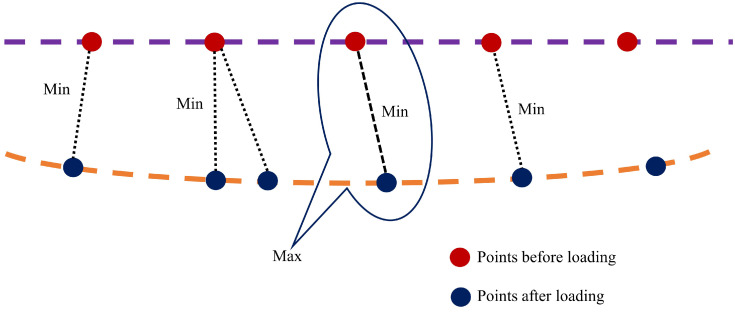
Schematic representation of Hausdorff distance measurement between points before and after loading the beam.

The Hausdorff distance is oriented asymmetrically, as well, which implies that $\delta_{h}\left(A,B\right)$ is always not equal to $\delta_{h}\left(B,A\right)$. The Hausdorff distance is dependent on the individual point cloud spatial position rather than the overall structure facade shape, which is perpendicular to the loading direction. The points acquired during loading and unloading serve as two sets of points for applying the Hausdorff distance mechanism, as shown in Figure 4.

### 3.3. Experimental Design

**Figure 5:**
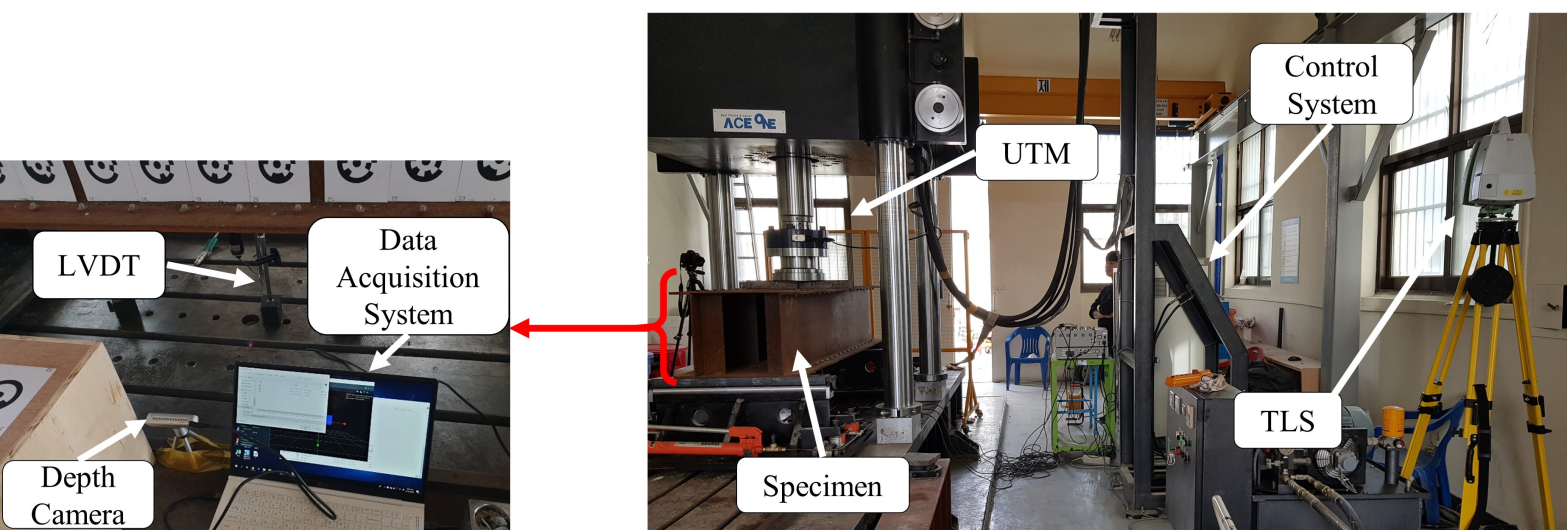
Experimental setup.

The proposed methodology results obtained in this study were verified by conducting indoor laboratory tests, as shown in Figure 5. The beam used for the laboratory test was a steel box girder, SS400-6T, with dimensions of 0.4 m × 0.8 m × 2 m. The load was applied at the center of the span using a universal testing machine (UTM). The specimen has 5 stiffeners 45 cm apart on each web entity for preventing lateral–torsional buckling. Four different loads were applied to attain the nominal deflection measured by the LVDT. First, the unloaded specimen was scanned using both sensors for obtaining reference data. Then, the specimen was scanned simultaneously using TLS and the DC immediately after loading and attaining nominal deflections of 1 mm, 2 mm, 3 mm, and 4 mm. The TLS field of view specification allowed us to scan the entire specimen during the experiment. However, this was not possible when using the DC because its field of view allowed us to scan of a limited area of the specimen. Therefore, we selected the most sensitive area of the specimen and scanned it using the DC. The bottom area around the mid-span where was considered the area most sensitive to flexural deflection. Therefore, we mounted the LVDT at the center of the span for validating the results.

## 4. Results and Discussion

### 4.1. Depth Camera Data Processing

**Figure 6:**
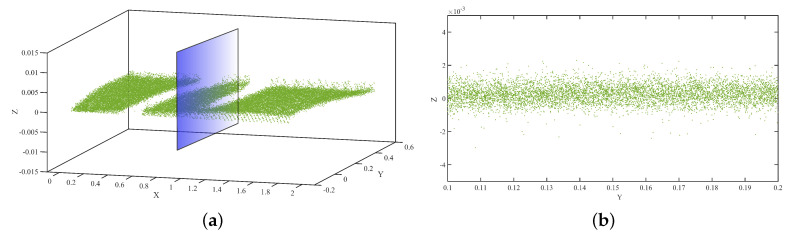
(**a**) Point cloud acquired from DC for the target area of the specimen; (**b**) section view.

**Figure 7:**
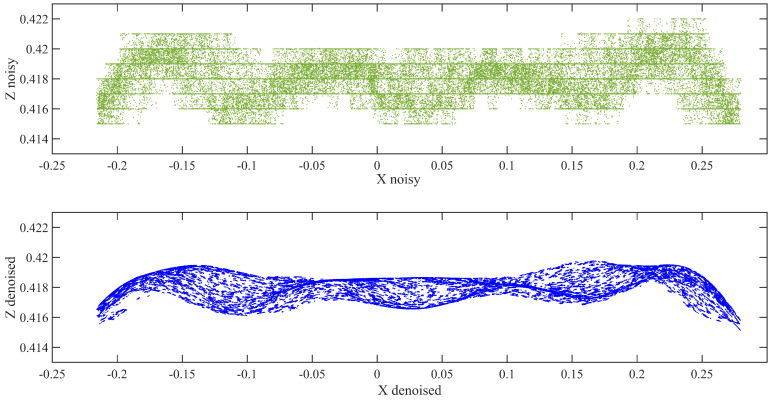
DC data before and after bilateral filtering.

The deflection of the beam was determined from the DC data using the following procedure:

- The D415 depth camera was fixed on the horizontal leveled area on the right at the bottom of the center along the span, as shown in Figure 5.
- All the scanning processes conducted in the same position. Figure 6 depicts the data acquired using the Intel RealSense D415 that were analyzed by changing the .bin file format first into the .pts and then into the .txt format, which enabled us to easily interpret the data using Cloud Compare and MATLAB software packages. The necessary pre-processing steps, such as removing unwanted data, downsampling the data, statistical outlier removal (SOR), and manual cropping of the farthest outliers, were performed in the Cloud Compare software using the .pts file format.
- Because the DC is very sensitive to inherent noise, noise should be treated prominently. Bilateral filtering techniques were applied based on the pseudocode described in Section 2.2.2, preceded by using the IQR score to minimize the noise in the data by eliminating outliers. The resulting point cloud of the scene after eliminating the outliers is shown in Figure 7.
- The proposed Hausdorff distance measurement algorithm was executed using the points obtained from the loading and unloading scenarios per Equation (3).
- Once we obtained the Hausdorff distance for each loading scenario, we compared it with the LVDT output.

### 4.2. Terrestrial Laser Scanning Data Processing

The beam deflection was measured from TLS data using the following method.

**Figure 8:**
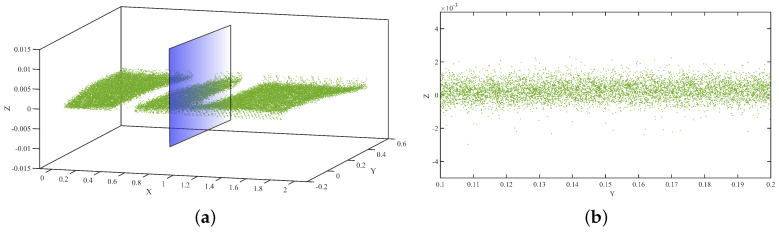
(**a**) Point cloud acquired via TLS, (**b**) section view.

**Figure 9:**
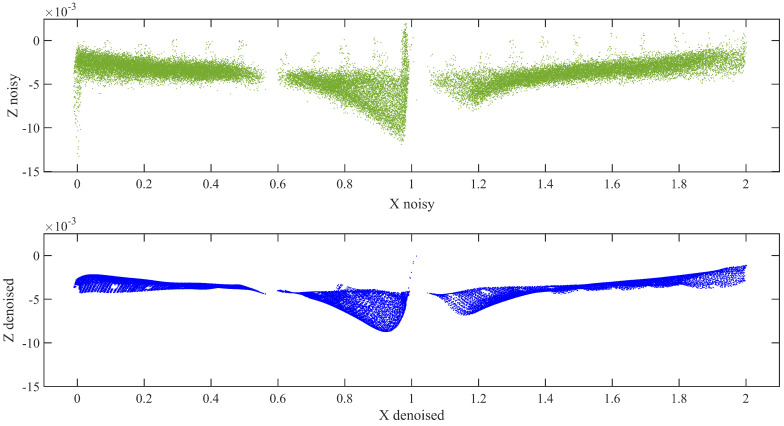
Terrestrial laser scanning (TLS) data before and after bilateral filtering.

- The Leica C5 scanner was placed 2.5 m away from the specimen during the laboratory experiment. The incident angle and range of a scanner are selected based on the factors affecting the accuracy of the data [39]. The scanning process was started immediately after applying the required load and attaining the LVDT reading for the nominal deflection. The acquired raw data form the scanner shown in Figure 8 was transformed into the .pts or .xyz file format, for easy analysis using Cloud Compare.
- The necessary pre-processing steps, including SOR, manual trimming, and segmentation were conducted for the raw data to decrease noise and increase accuracy. According to this approach, the bottom flange was more effective in determining the deflection. Therefore, our target was to tear out the bottom flange during segmentation.
- Similar to the analysis of the DC data, the bilateral filtering techniques were also applied to the TLS data for thorough removal of noises, as shown in Figure 9.
- Once we obtained a clear representative of the specimen point cloud data, we employed the Hausdorff distance approach for the loading and unloading data separately. We then Compared and validated these results with those obtained using the DC and LVDT sensors.

The data obtained from the TLS and DC showed different qualities during processing. The data obtained from the DC had a 7-mm-thick layered set of points with 1 mm vertical spacing, as shown in Figure 6a. However, there is a significant vertical gap between the points, which indicates the depth of a scene when capturing the specimen image. Specifically, in our experiment, this depth difference was the main challenge for achieving our target. This depth variation is generated owing to the nature of the specimen, environmental factors, insignificant color variation along the surface of the specimen, and so on. In contrast, in Figure 8b, although the data look scattered and do not have layered set of points as observed in the DC data, the thickness and vertical spacing between the points are very small. In addition, in our analyses, the vertical variation accuracy is significant because we compared the vertical change in position (deflection).

### 4.3. Validated Result and Comparison

**Figure 10:**
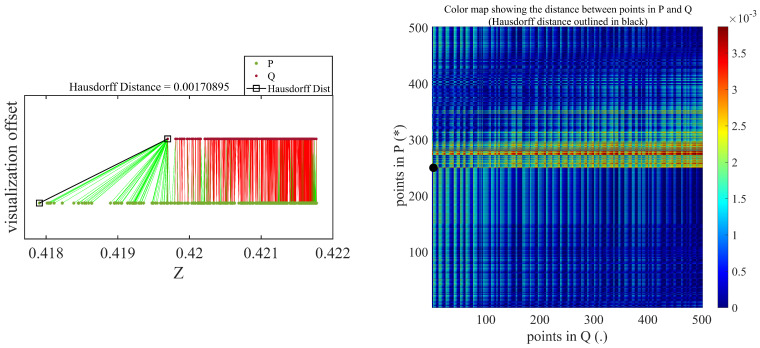
Hausdorff distance measurement result for 1-mm nominal deflection using depth camera.

**Figure 11:**
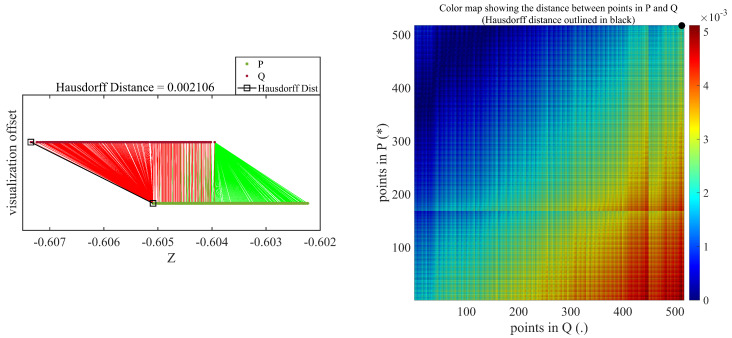
Hausdorff distance measurement result for 1-mm nominal deflection using terrestrial laser scanner.

Figure 10 and Figure 11 show the Hausdorff distances measured between deflected and undeflected beams using the DC and TLS datasets, respectively. These results are only for a nominal deflection of 1 mm. As discussed in Section 4.1, the densified concentration of points in the vertical direction can be observed in the TLS Hausdorff distance measurements. There are numerous points relative to the DC, as shown in Figure 11. The color map are shown on the right side of Figure 10 and Figure 11, illustrating the distance between all individual constituents of a set of points in Q (assigned as deflected) and a set of points in P (assigned as undeflected).

Based on the methodology depicted in Figure 3, outputs from the two devices were compared. The TLS and DC outputs improved with the increasing nominal deflection. As mentioned in the discussion on pre-processing, to diminish the error resulting from manual cropping of the region of interest (RoI), repeatability of experiments and having multiple datasets for a specific measurement are very crucial. As manual trimming was unavoidable in the pre-processing step, we used the same data multiple times but different pre-processing for each load case of dataset. Therefore, we analyzed the data nine times for a single measurement and then took the average. Consequently, we obtained 4 × 9 = 36 outputs for one case in the dataset of each sensor. For each sensor, we obtained noised and denoised data. This yielded 144 outputs. Through repeating this analysis for each dataset, we could decease the effects of the errors resulting from pre-processing. For clarity and conciseness, Table 2 presents only the averaged value of each flexural deflection measurement using the TLS and DC along with the LVDT. However, we present the nine outputs analyzed for a 4-mm nominal deflection in the denoised measurement in Table 3.

**Figure 12:**
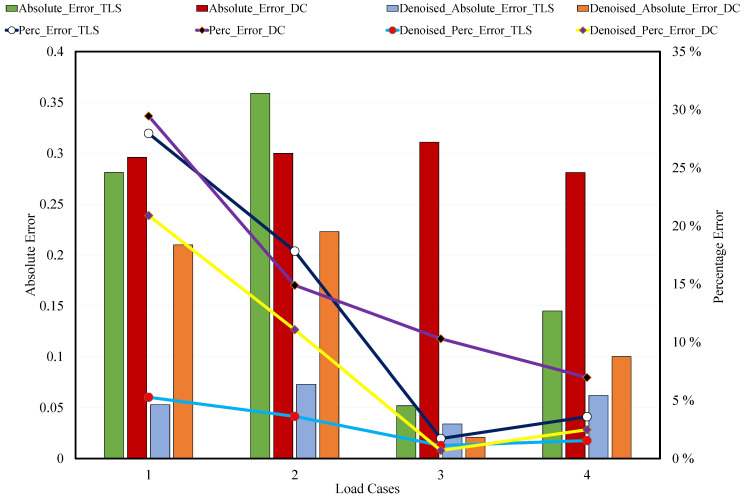
Comparison of absolute and percentage errors of TLS and DC with linear variable differential transformer (LVDT).

The error percentages and absolute errors of the TLS and DC measurements for each load case before and after denoising are presented graphically in Figure 12. The percentage error is expressed as the percentage of the difference between the measured value (proposed result) and the known value (LVDT reading), whereas the absolute error defines the gap between the measured and known values. According to the results in Table 2, even the LVDT measurements have slight errors, which are acceptable. Fortunately, the error graph for the TLS and DC measurements is a decreasing function with respect to the nominal deflection. As shown in Figure 7 and Figure 9, the errors in the TLS and DC results were enhanced after bilateral filtering. However, both devices showed better outputs after bilateral filtering. The percentage errors of 29.45%, 14.90%, 10.29%, and 6.97% in the DC outputs before denoising improved to 20.90%, 11.07%, 0.69%, and 2.48% for nominal deflections of 1 mm, 2 mm, 3 mm, and 4 mm, respectively. Similarly, the percentage errors of 27.96%, 17.83%, 1.72%, and 3.60% in the TLS outputs before denoising improved to 5.27%, 3.62%, 1.13%, and 1.54% for 1 mm, 2 mm, 3 mm, and 4 mm, respectively. Therefore, we can conclude that the denoising technique is effective for data from both the sensors but more effective for the TLS data than for the DC data. Generally, after denoising the data acquired by the two sensors, percentage errors of 5.27%, 3.62%, and 1.54% obtained in the TLS data for nominal deflections of 1 mm, 2 mm, and 4 mm, respectively, lower than those obtained in the DC data. However, the percentage error of 0.69% for the 3-mm nominal deflection obtained in the DC data is lower than that observed in the TLS data. The error percentages for 1- and 2-mm nominal deflections observed in the DC data are beyond 10% and much higher than those for the remaining nominal deflections. This indicates that the measurements of 1- and 2-mm nominal deflections obtained via the DC are not acceptable based on the specific setup and environment conditions that we used in the experiment; nevertheless the remaining measurements, at 3- and 4-mm nominal deflections, are satisfactory. However, the TLS results for all nominal deflections are acceptable and very satisfactory. In addition, both devices showed better and accurate outputs for the 3-mm nominal deflection, whereas the LVDT measured 3.022 mm. As expected, we can conclude that the TLS provides better outputs than the DC overall. For the 3- and 4-mm nominal deflections, we have shown that, when using a robust denoising technique on the datasets, the inexpensive DC provides satisfactory deflection measurements, but its performance is inferior to that of the more precise and expensive TLS.

## 5. Conclusions

Vision-based sensors have a considerable impact on structural health monitoring because of their high-speed data acquisition performance, ability to scan real-time structural 3D shapes, capacity to obtain measurements from inaccessible sites conditions, portability, and so on. However, all these devices have their own limitations, which depend on their function. A fundamental limitation when using a DC for determining the deformation of a structure is its narrow field of view. This prohibits us from visualizing the overall shape of a structure, unless many different camera setups are used, followed by registration data processing, which is time consuming and tedious. Therefore, the proposed methodology can be used only on the critical areas of a structure. A TLS is also very sensitive to noise, which is generated either from the inherent nature of the device or the environment. Researchers have been trying to improve accuracy using different denoising and filtering methods, as well as robust algorithms, to obtain the desired output.

This study comprehensively analyzed the performance of an inexpensive vision-based sensor and compared it with a highly precise but very expensive sensor. In addition, this study explains the effect of using bilateral filtering on sensor data to enhance structure health monitoring. Accordingly, the deflection of a beam was estimated based on the same approach—the Hausdorff distance measurement—using data obtained from the TLS and DC. Bilateral filtering techniques were also applied to data from both sensors to obtain better outputs. 

## Figures and Tables

**Table 1:** Technical specifications of Intel RealSense D415 Depth Camera and Leica Terrestrial Laser Scanner.

Parameter	Terrestrial Laser Scanner	Depth Camera
Brand	Leica	Intel RealSense
Model	C5	D415
Range	300 m @ 90 %; 134 m@ 18 % albedo (minimum range 0.1 m)	∼10 m
Field of View (H × V)	360° × 270°	69.4° × 42.5° × 77°
Range measurement principle	Pulsed (Time of Flight)	Active IR Stereo
Scan rate	50,000 points/s	-
Resolution	-	1280 × 720
precision	2 mm	-
Baseline	-	55 mm
Point spacing	Fully selectable horizontal and vertical; <1 mm minimum spacing, through full range; single point dwell capacity.	-
IR Projector	-	Standard
Camera	Auto adjusting, integrated high-resolution digital camera with zoom video	Full HD RGB camera calibrated and synchronized with depth data

**Table 2:** Summary of deflection measurements obtained with both vision-based sensors.

			TLS		Percentage Error		DC		Percentage Error	
Nominal Deflection (mm)	LVDT 1.00 m (mm)	Loading (KN)	Noised mm	Denoised mm	Error Noised %	Error Denoised %	Noised mm	Denoised mm	Error Noised %	Error Denoised %
Unloading	0	0	0	0	0	0	0	0	0	0
1	−1.005	57.33	−1.286	−1.058	27.96	5.27	−0.709	−0.795	29.45	20.90
2	−2.014	200.85	−2.373	−2.087	17.83	3.62	−1.714	−1.791	14.90	11.07
3	−3.022	380.85	−2.970	−2.988	1.72	1.13	−2.711	−3.043	10.29	0.69
4	−4.029	480.84	−4.174	−3.967	3.60	1.54	−4.310	−4.129	6.97	2.48

**Table 3:** Deflection measurements obtained through repeated analysis of denoised datasets in both sensors for a 4-mm nominal deflection.

Nominal Deflection (mm)	LVDT 1.00 m (mm)	Loading (KN)	TLS_Denoised									DC_Denoised								
			Case									Case								
			1	2	3	4	5	6	7	8	9	1	2	3	4	5	6	7	8	9
Unloading	0	0	0	0	0	0	0	0	0	0	0	0	0	0	0	0	0	0	0	0
4	−4.029	480.84	3.82	3.79	3.98	4.07	3.98	4.04	3.88	4.02	4.13	4.43	4.29	4.27	4.13	4.07	4.08	3.98	3.99	3.92
Average			−3.9667									−4.1295								

## Data Availability

Data sharing not applicable.

## Abbreviations

The following abbreviations are used in this manuscript:

3D	Three-Dimensional
LIDAR	Light Detection and Ranging
TLS	Terrestrial Laser Scanning
DC	Depth Camera
LVDT	Linear Variable Differential Transformer
IQR	Inter Quartile Range
RGB	Red Green Blue
ToF	Time of Flight
UTM	Universal Testing Machine
